# EasyBOPT: A Digitally Standardized Protocol to Simplify and Reproduce the Biologically Oriented Preparation Technique (BOPT)—Technique Description and Case Series

**DOI:** 10.3390/jcm15072591

**Published:** 2026-03-28

**Authors:** Rubén Agustín-Panadero, Ignazio Loi, Ana Roig-Vanaclocha, José Amengual-Lorenzo, César Chust-López, Marina García-Selva, Miguel Gómez-Polo, Blanca Serra-Pastor

**Affiliations:** 1 Department of Stomatology, Faculty of Medicine and Dentistry, University of Valencia, 46010 Valencia, Spain; ruben.agustin@uv.es (R.A.-P.); aroigva@hotmail.com (A.R.-V.); ppamengual@gmail.com (J.A.-L.); marina.garcia@uv.es (M.G.-S.); 2 Private Practice, 09125 Cagliari, Italy; loi.ig@tiscali.it; 3 Corus Chust Dental Laboratory, 46008 Valencia, Spain; cesarchust@gmail.com; 4 Department of Stomatology, Faculty of Dentistry, Universidad Complutense de Madrid, 28040 Madrid, Spain; mgomezpo@ucm.es

**Keywords:** BOPT, EasyBOPT, eBOPT, vertical preparation, digital provisionalization, dental restorations, gingival health

## Abstract

**Background**: The Biologically Oriented Preparation Technique (BOPT) protocol has demonstrated excellent clinical and biological outcomes in restorations with vertical preparation designs. However, its provisional phase remains highly dependent on the operator’s skill. The EasyBOPT (eBOPT) protocol introduces a standardized digital workflow aimed at simplifying this process and enhancing its reproducibility. The primary objective of this study is to describe the eBOPT protocol and to compare its clinical outcomes with the conventional BOPT approach in a prospective case series. **Materials and Methods**: A case series was conducted including ten patients requiring full-coverage restorations in the maxillary anterior region. The study protocol was registered on 15 July 2024 at ClinicalTrials.gov, with the following registry name: Periodontal outcomes and digital volumetric variation following BOPT restorations, and with the following registration number: NCT06485999. Five patients were treated using the conventional BOPT protocol and five with the eBOPT protocol. Both groups followed identical diagnostic, preparation, and definitive restoration phases, differing only in the provisionalization technique. In the eBOPT group, a digital workflow based on dual scanning (physiologic and retracted gingiva), “best-fit” alignment, and CAD-CAM fabrication of a standardized 45° emergence provisional restoration was employed. Operative time per tooth, gingival healing time, and the need for additional adjustments were recorded. **Results**: The mean clinical time per tooth was significantly lower with the eBOPT protocol (12.30 ± 1.50 min) compared to the conventional protocol (31.20 ± 2.10 min; *p* < 0.001). The mean gingival healing time was also reduced with eBOPT (4.4 ± 0.9 weeks) relative to the traditional approach (9.6 ± 1.5 weeks; *p* = 0.0014). Only the conventional group required additional provisional adjustments (80% of cases; *p* = 0.048). **Conclusions**: The EasyBOPT protocol enables faster, more predictable, and less operator-dependent provisionalization while maintaining the biological stability and gingival health achieved with conventional BOPT. This digital workflow simplifies the clinical application of the BOPT concept and enhances its reproducibility.

## 1. Introduction

The Biologically Oriented Preparation Technique (BOPT), introduced by Loi in 2013 [1], represented a paradigm shift in tooth preparation for full-coverage restorations. Unlike conventional preparations featuring a chamfer or shoulder, BOPT eliminates the horizontal finish line and replaces it with a vertical preparation accompanied by rotary gingitage. The objective of this procedure is to create a controlled biological bed capable of stabilizing the intrasulcular blood clot and promoting soft tissue regeneration. This approach not only simplified tooth preparation geometrically but, more importantly, established a new biological framework in which the gingival margin and prosthetic emergence profile are determined by the interaction between the provisional restoration and the periodontal tissue response.

The biological principles underlying BOPT are based on two well-documented phenomena in the literature [2,3,4,5,6,7]: creeping attachment and mechanotransduction. The former refers to the gingival tissue’s ability to migrate coronally and adapt to the new convex contour generated by the provisional restoration, resulting in biotype thickening and improved marginal stability. The latter, mechanotransduction, describes the adaptive response of gingival tissue to the mechanical and functional stimuli exerted by the prosthesis, promoting its differentiation and long-term consolidation. Thus, the provisional restoration is not merely conceived as a temporary replacement but as a therapeutic instrument that guides and shapes the soft tissue throughout the healing period.

Over the past decade, cumulative clinical evidence has confirmed the efficacy and predictability of the BOPT protocol. In a two-year prospective trial, Agustín-Panadero [8] demonstrated that zirconia BOPT restorations fabricated through a fully digital protocol maintain stable gingival margins, prevent the occurrence of recessions, and promote significant soft tissue thickening both buccally (0.49 mm) and palatally (0.39 mm), with stable periodontal indices and minimal incidence of biological complications. Furthermore, in a ten-year prospective follow-up study [9] on zirconia restorations fabricated from conventional impressions, the same authors confirmed that these tissue modifications remain stable over time, regardless of the impression technique used. The study reported a significantly lower rate of gingival recession in BOPT-treated teeth (7.7%) compared to untreated antagonist teeth (38.4%), with a survival rate of 97.8% and a success rate of 94.5%. These findings consolidate BOPT as a reliable and biologically respectful alternative for the restoration of anterior teeth.

Despite its biological and clinical advantages, the conventional BOPT protocol presents certain technical limitations during the provisional phase. Traditionally, the provisional restoration is fabricated by the dental laboratory as an ultrathin “eggshell” (0.2 mm) prior to tooth preparation, which is subsequently relined intraorally. This relining, along with the specific contour modifications performed by the clinician, generates the subgingival emergence profile. Consequently, the provisional restoration must be designed, polished, and manually adjusted by the operator, resulting in extended chair time. As noted by Paniz [10], Serra [11], and Agustín-Panadero [12], this phase is critical, as proper gingival healing and long-term stability depend on it; moreover, it is highly operator-dependent and introduces clinical variability according to the clinician’s experience. Likewise, the conventional BOPT provisional may require multiple adjustment appointments during the 8–12 weeks following placement, particularly in cases requiring gingival margin remodeling or leveling. In summary, while the conventional protocol performs predictably, it demands substantial manual skill and clinical experience, which may limit its reproducibility in less experienced hands or in educational settings.

With the rise of digital dentistry, several approaches [13] have been proposed to reduce operator-dependent variability. Among these, the use of digital tools for planning emergence profiles or controlling the invasion of definitive restorations into the periodontal tissues has gained particular relevance. However, to date, the provisional process has continued to rely largely on the clinician’s manual modeling.

In this context, the present study introduces the EasyBOPT (eBOPT) protocol, a digital variant of the BOPT that preserves the biological principles of the original technique while simplifying and standardizing the critical provisional phase. By combining dual intraoral scanning (physiologic and retracted gingival positions), digital alignment of STL files, and CAD-CAM design of provisional restorations, the eBOPT protocol eliminates the need for manual modeling, reduces inter-operator variability, and shortens the operative and healing time.

The primary objective of this study is to describe the eBOPT protocol and to compare its clinical outcomes with the conventional BOPT protocol in a prospective case series. Specifically, the following parameters were evaluated: periodontal parameters, total operative time, provisionalization time, gingival healing time, and short-term stability of the gingival margin. The working hypothesis is that the eBOPT protocol will significantly reduce clinical time and operator variability while maintaining the biological efficacy demonstrated by the conventional BOPT.

## 2. Materials and Methods

This clinical investigation was designed as a case series involving ten patients (n = 10) treated at the Clínica Odontológica Lluís Alcanys, University of Valencia. The study protocol was registered in 15 July 2024 at ClinicalTrials.gov, with the following registry name: Periodontal outcomes and digital volumetric variation following BOPT restorations, and with the following registration number: NCT06485999. This research was approved by the Ethic Committee for Research Involving Human Subjects (2024-ODON-3325009) of the University of Valencia in Spain (9 May 2024). No participants were enrolled and no clinical procedures were initiated before Ethics Committee approval and ClinicalTrials.gov registration were obtained. The Declaration of Helsinki for experiments involving human subjects was adhered to, and all patients provided their informed consent to take part in the trial by signing an informed consent form. All patients required full-coverage restorations in the maxillary anterior region, ranging from the right canine (tooth 13) to the left canine (tooth 23), with the number of restorations varying from one to six per patient depending on individual needs. Prior to treatment, patients were randomly divided into two groups of five subjects each using a web-based randomization tool (www.random.org): a control group treated with the conventional BOPT protocol and an experimental group treated with the new eBOPT protocol. All clinical procedures were performed by a single operator (R.A.-P.), who had extensive experience with the BOPT, in order to minimize execution-related variability. Operative times and gingival healing periods were recorded for each case.

**Figure 1:**
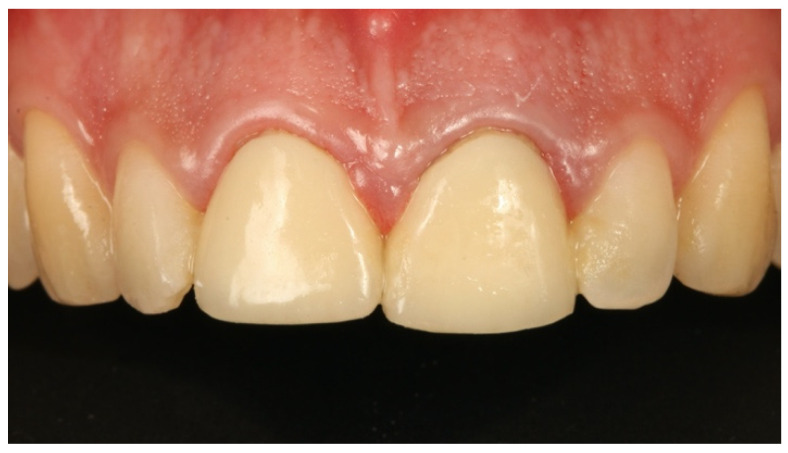
Frontal view of the initial situation. Old restorations are observed on the maxillary central incisors (teeth 11 and 21), showing periodontal alteration and mucogingival defects that caused gingival recession due to poor marginal adaptation.

Inclusion criteria were as follows: patients over 18 years of age; presence of existing full-coverage restorations in the maxillary anterior region with an indication for replacement (Figure 1); gingival tissues with no bleeding on probing and probing depths ≤ 3 mm; adequate motivation; and good oral hygiene control. Exclusion criteria included active periodontitis or advanced bone loss, systemic diseases that could compromise healing, tobacco consumption exceeding ten cigarettes per day, and pregnancy or lactation.

The initial clinical protocol was identical for all patients and included anamnesis, intraoral and extraoral examination, and treatment planning. In each case, a double periodontal probing was performed to determine the position of the cementoenamel junction (CEJ) and the alveolar crest. Based on these measurements, the vertical limit of the tooth preparation was established approximately 1 mm coronal to the bone crest, which in most cases corresponded to about 1 mm apical to the CEJ. The margin of the provisional restoration was systematically positioned 2 mm coronal to the bone crest, thus ensuring preservation of the supracrestal attached tissue.

**Figure 2:**
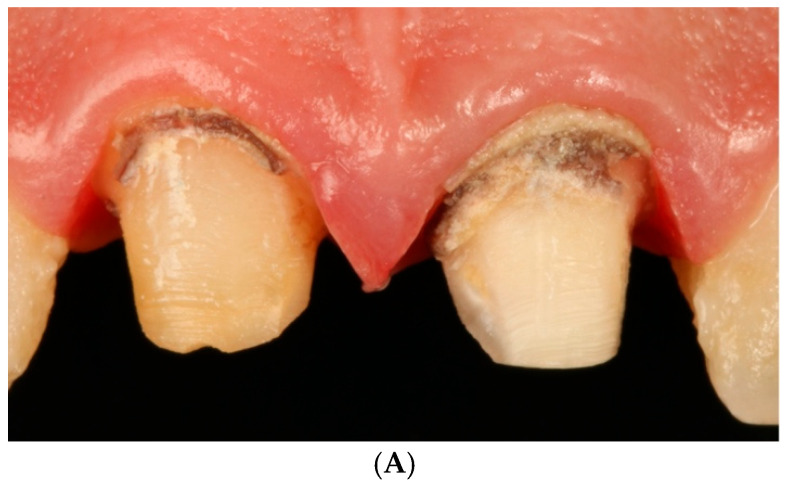

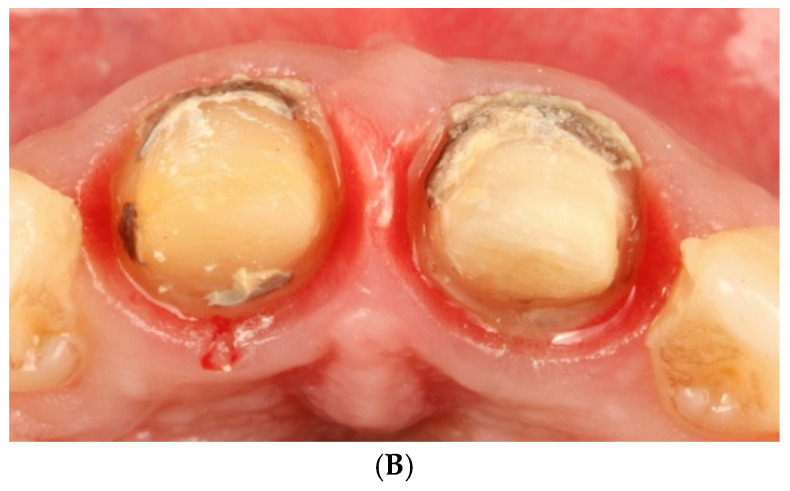
(**A**) Frontal view after removal of the old restorations. Secondary caries are evident on both abutment teeth as a consequence of the previous marginal misfit. (**B**) Occlusal view of the abutment teeth after removal of the restorations. Secondary caries and plaque accumulation are evident due to the defective marginal seal.

**Figure 3:**
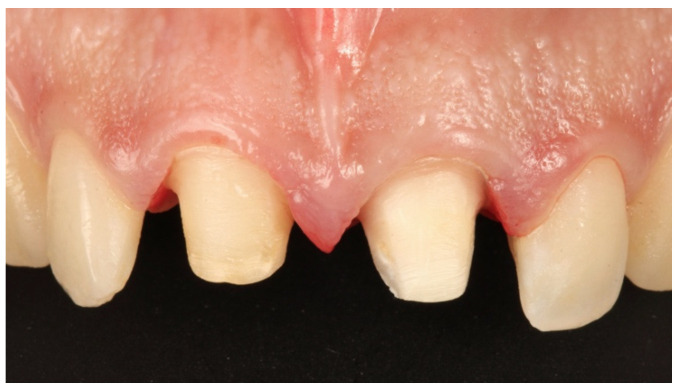
Abutment teeth after cleaning and polishing of the dentin surfaces.

Tooth preparation was performed following the principles of the BOPT protocol, identically in both groups, using the simplified BOPT preparation technique described by Agustín-Panadero and Solá [14]. Starting from a pre-existing finish line (Figure 2 and Figure 3), an intrasulcular preparation was carried out using a specific diamond bur (LM863.125.012, BOPT drills, Leader Medica, Padua, Italy) through three sequential phases with different angulations: (1) an initial opening and gingitage phase with a 10–15° inclination relative to the tooth axis, intended to open the sulcus by de-epithelializing its internal wall and removing the natural cervical emergence of the tooth; (2) a second phase to eliminate the pre-existing finish line, with the bur parallel to the longitudinal axis (0°); and (3) a final convergence and polishing phase with a 3–6° angulation, repeating the sequence with burs of decreasing grit until a homogeneous surface was obtained.

During the preparation, both the free and attached gingival epithelium were simultaneously de-epithelialized, detaching the periodontal tissue until reaching the connective layer (gingitage). This procedure created a controlled bleeding bed capable of stabilizing the clot and guiding gingival remodeling through the use of a PMMA provisional restoration for a period of 4 to 8 weeks.

**Scheme 1:**
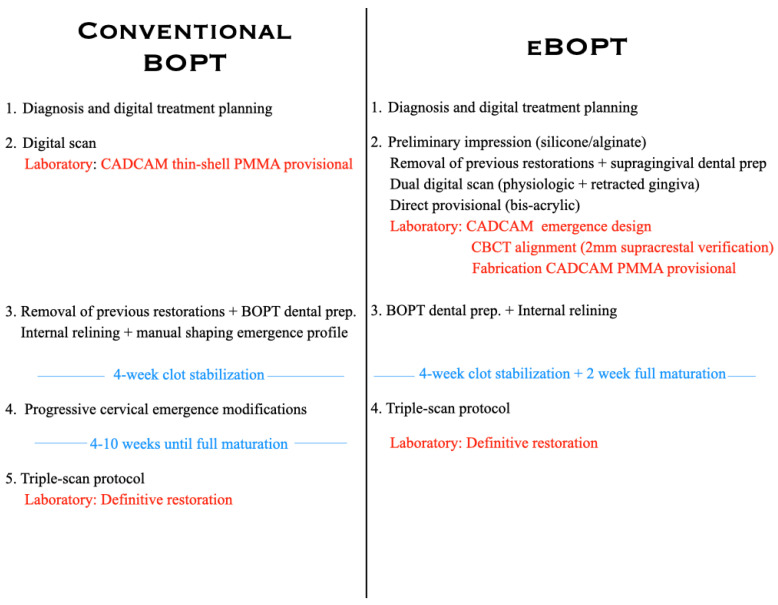
Comparative graphical abstract of conventional BOPT and eBOPT workflows.

The provisional phase represented the main difference between the two protocols (Scheme 1). In the conventional protocol, a preliminary digital scan was taken at the first appointment, before removing the existing crowns, and sent to the dental laboratory for fabrication of an “eggshell” PMMA (Sintodent, Roma, Italy) provisional restoration (approximately 0.2 mm in thickness). At the second appointment, once the pre-existing crowns were removed (Figure 2 and Figure 3), the BOPT vertical preparation was performed as previously described, and the PMMA eggshell was relined intraorally with acrylic resin (Sintodent). After relining, the cervical emergence profile of the provisional restoration was manually shaped following the technique described by Loi [1]. This technique recommends that the initial provisional should present an emergence angle between 30 and 45°, and after four weeks of healing, the emergence can be modified to achieve gingival margin leveling or remodeling when necessary. The provisional restoration was cemented with a temporary cement (Temp Bond, Kerr, Orange, CA, USA), left in place for four weeks, and subsequently adjusted in cases requiring gingival margin refinement until the desired contour was achieved.

**Figure 4:**
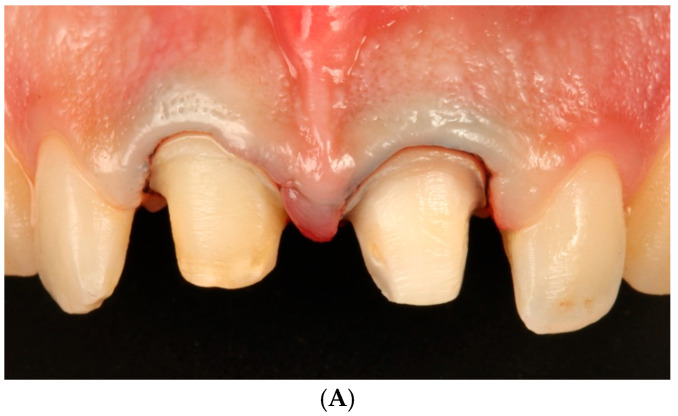

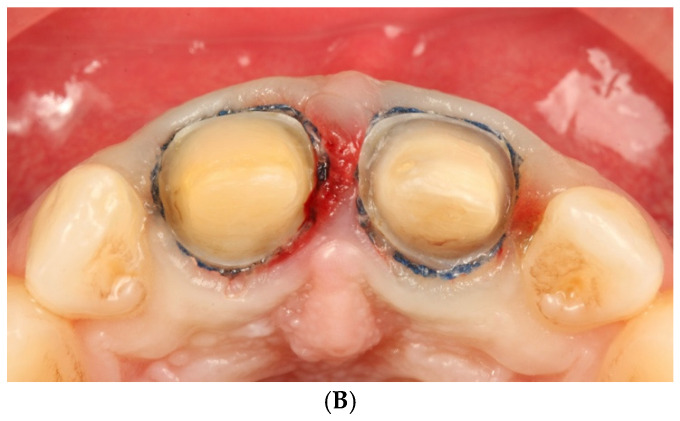
(**A**) Frontal view of the abutment teeth with a chamfer finish line. Gingival retraction was achieved using the double retraction cord technique (size 1). (**B**) Occlusal view showing gingival retraction obtained with the double-cord technique. Both teeth display proper taper and a well-defined chamfer margin.

**Scheme 2:**
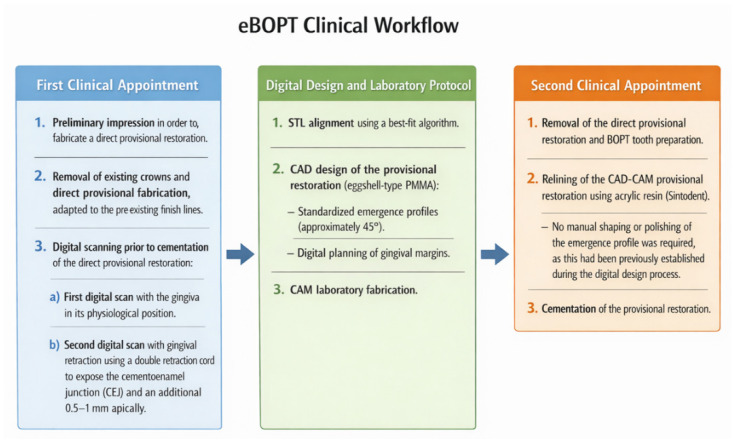
Schematic overview of the eBOPT clinical and laboratory workflow.

In the eBOPT protocol (Scheme 2), the clinical workflow was divided into several stages. At the first appointment, a preliminary conventional impression was taken without removing the existing crowns in order to use it as a print to fabricate a direct provisional restoration. The second step in this same appointment was the removal of the old crowns, and after that, the conventional impression was used to fabricate a direct provisional restoration made of bis-acrylic resin (Structur, Voco, Cuxhaven, Germany), adapted to the pre-existing finish lines of the abutment teeth (Figure 3). Prior to cementation of this direct provisional, the specific digital protocol for eBOPT was performed. This consisted of an initial digital scan (Marathon MY-1000, Padua Italy) of the tooth with the gingiva in its physiological position, followed by a second scan after placing a double retraction cord #1 (Ultrapak Cleancut, Ultradent, Salt Lake city, UT, USA) (Figure 4), thereby exposing the CEJ and an additional 0.5–1 mm apically.

**Figure 5:**
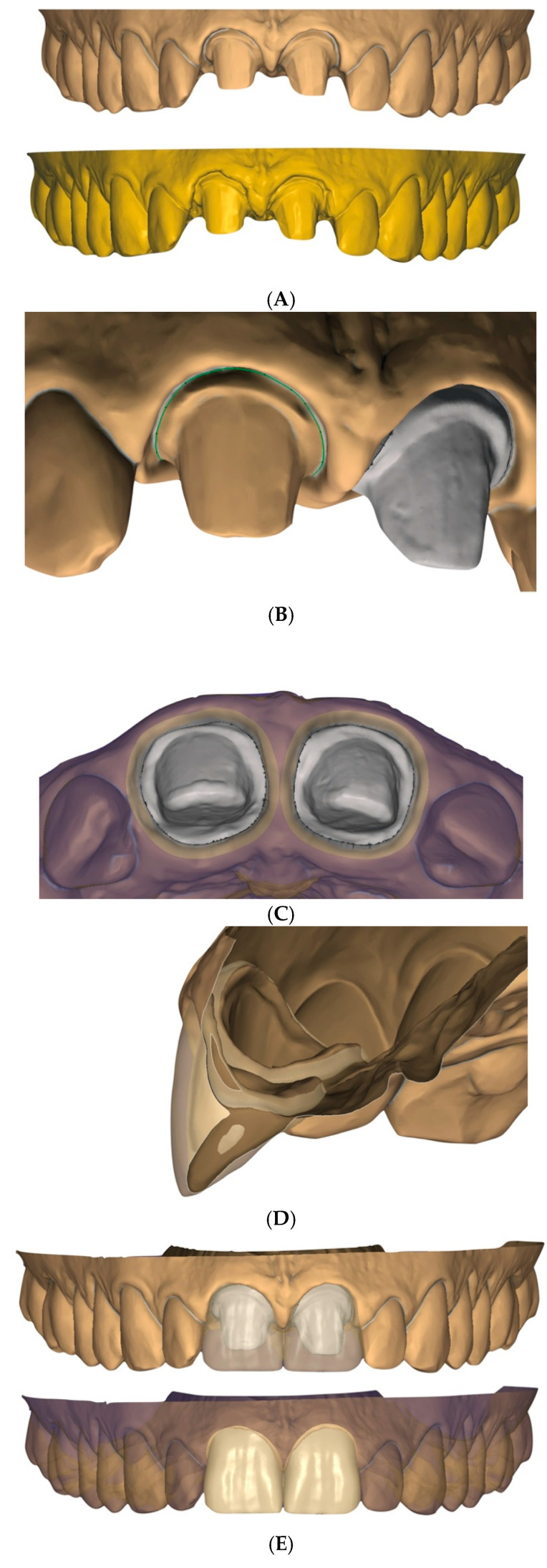

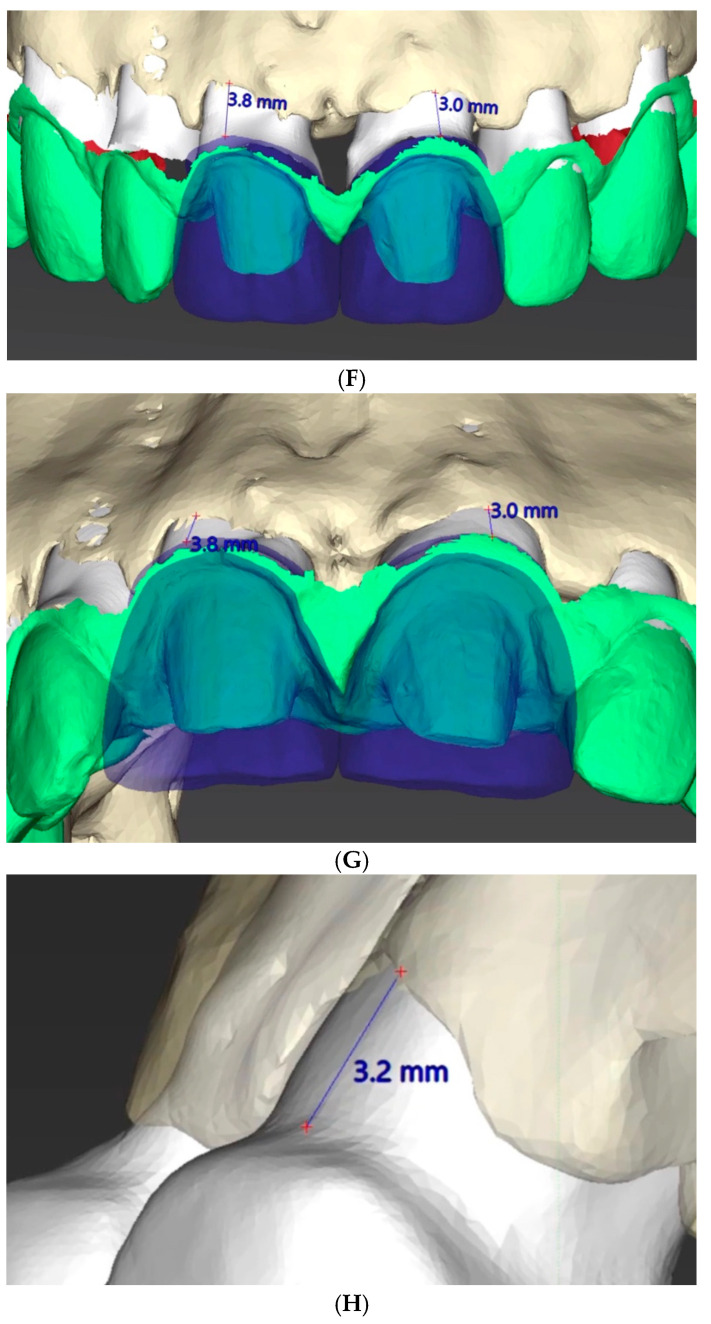
(**A**) Digital image of the STL file obtained after gingival retraction. The master model shows an accurate gingival contour that allows planning of the provisional restorations and the eBOPT technique. (**B**) Digital planning of the prosthetic finish line of the provisional restoration, positioned approximately 1 mm apical to the pre-existing margin, from which the new prosthetic emergence profile will originate. (**C**) Occlusal view of the digital design showing the peripheral contour of the provisional restorations during CAD fabrication. (**D**) Sagittal view illustrating controlled sulcular invasion in the design of the provisional restorations for both central incisors. (**E**) General view of the digitally designed provisional restorations. The individual morphology of each central incisor and the precise marginal adaptation at the retracted gingival area can be appreciated. (**F**,**G**) STL–DICOM alignment for biological validation of the emergence profile. The digitally designed wax-up of the provisional restoration (STL) was superimposed onto the CBCT dataset (DICOM) to evaluate the three-dimensional relationship between the prosthetic cervical emergence and the underlying alveolar crest. This digital verification enables measurement of the supracrestal distance between the most apical point of the emergence profile and the bone crest, confirming preservation of the biologically safe zone prior to fabrication of the CAD-CAM provisional restoration. (**H**) Verification of the supracrestal tissue dimension through STL–DICOM superimposition. This procedure allows confirmation that the cervical emergence of the restoration does not invade the supracrestal tissue dimension, maintaining a safety distance of approximately 2 mm from the bone crest.

**Figure 6:**
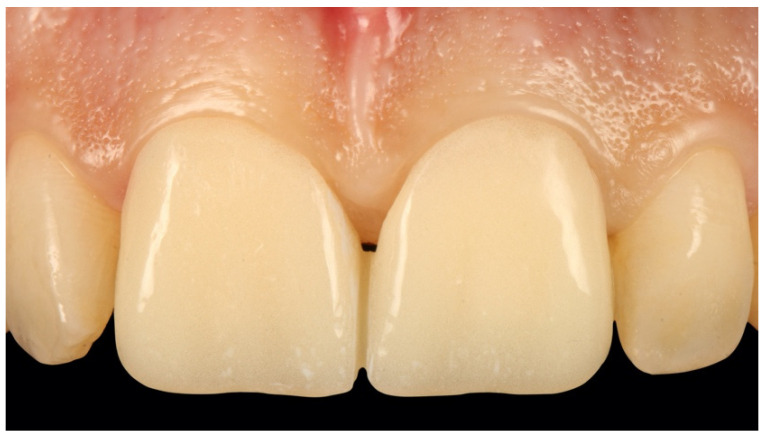
Clinical adjustment of the provisional restoration fabricated with the eBOPT protocol before the definitive preparation of the abutment teeth using the BOPT.

These two STL files were then aligned using a best-fit algorithm, allowing the laboratory to obtain both physiological and retracted gingival information. Based on this digital data, a CAD-CAM-fabricated “eggshell-type” PMMA provisional restoration (0.2 mm thick) was designed, featuring standardized emergence profiles (approximately 45°) and digitally preplanning the gingival margins using CAD software (ExoCAD 3.2, Align technology, Darmstadt, Germany). To further enhance biological safety and validate the digital design, the aligned STL files were additionally superimposed with the corresponding DICOM file obtained from CBCT imaging of the treated region. This three-dimensional alignment enabled precise visualization of the relationship between the designed cervical margin, the root surface, and the alveolar crest. The distance between the most apical point of the digitally designed emergence and the bone crest was digitally measured to confirm preservation of a minimum 2 mm supracrestal tissue dimension prior to CAD-CAM fabrication. This verification step complements the mechanical safety provided by the use of double size #1 retraction cords, which not only expose the subgingival area but also act as a physical safeguard maintaining a biologically safe distance from the alveolar crest during digital planning (Figure 5). This provisional and all its surfaces were milled and perfectly polished in the laboratory (Figure 6).

**Figure 7:**
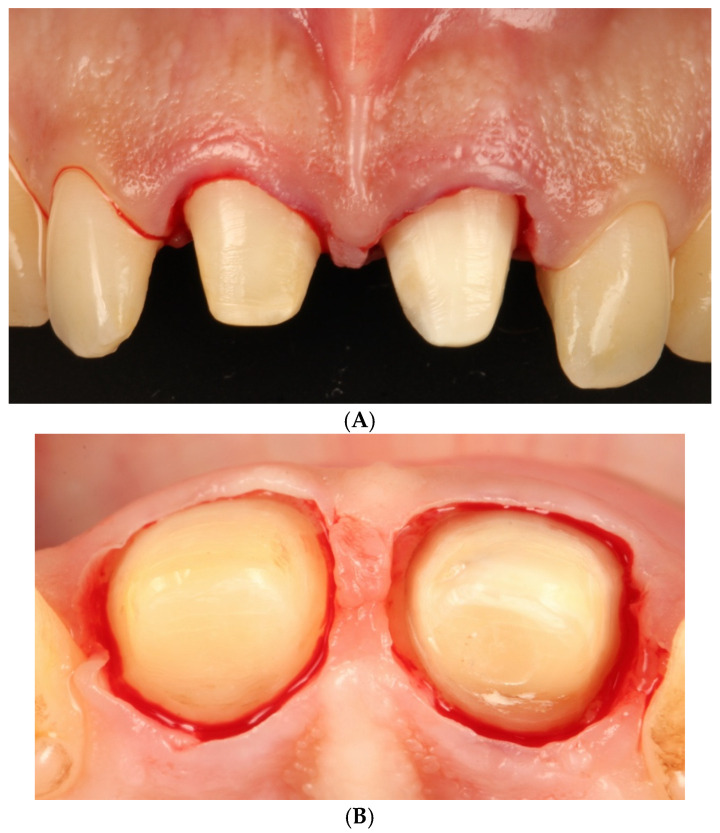
(**A**) Frontal view of the abutment preparation using the BOPT. The pre-existing finish line was completely removed, and rotary gingitage (gingival curettage) was performed. (**B**) Occlusal view of the BOPT preparations showing controlled de-epithelialization of the surrounding gingiva and shaping of the soft tissues.

**Figure 8:**
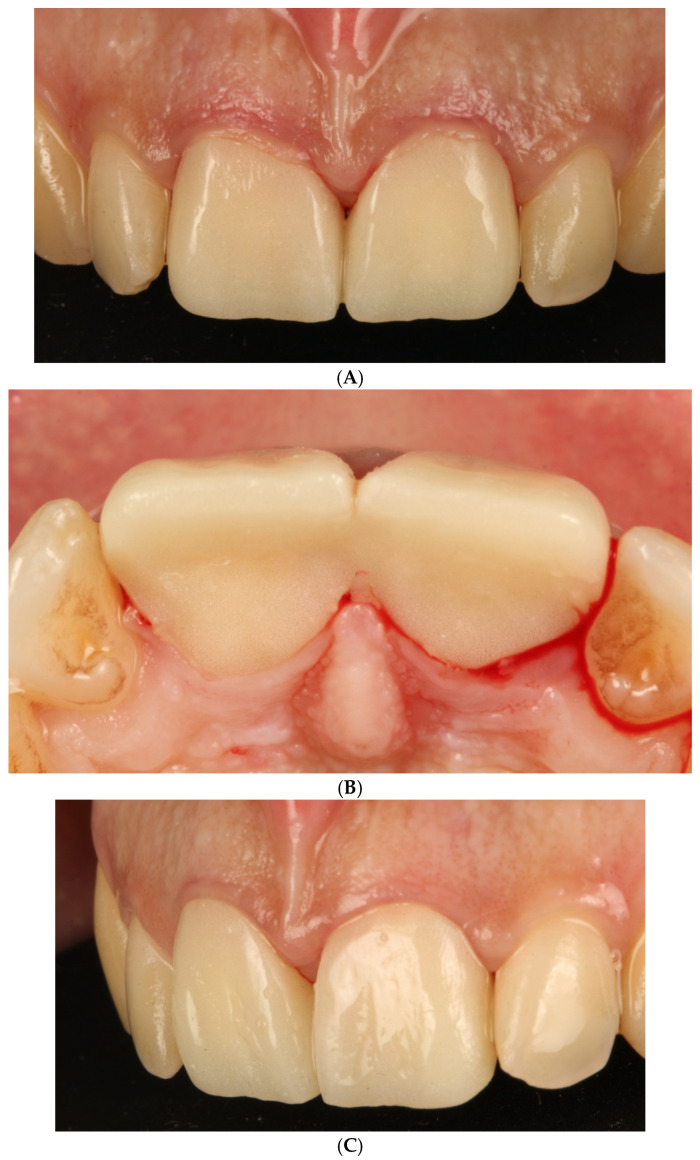
(**A**) Cementation of the provisional restoration after internal relining with PMMA acrylic resin of the initial eBOPT provisional. (**B**) Occlusal view of the provisional restoration cemented on the teeth prepared with the BOPT, showing accurate marginal adaptation and peripheral sealing. (**C**) Lateral view of the provisional restoration cemented on the BOPT abutment teeth, showing proper cervical emergence that allows leveling of the gingival margins of teeth 11 and 21.

**Figure 9:**
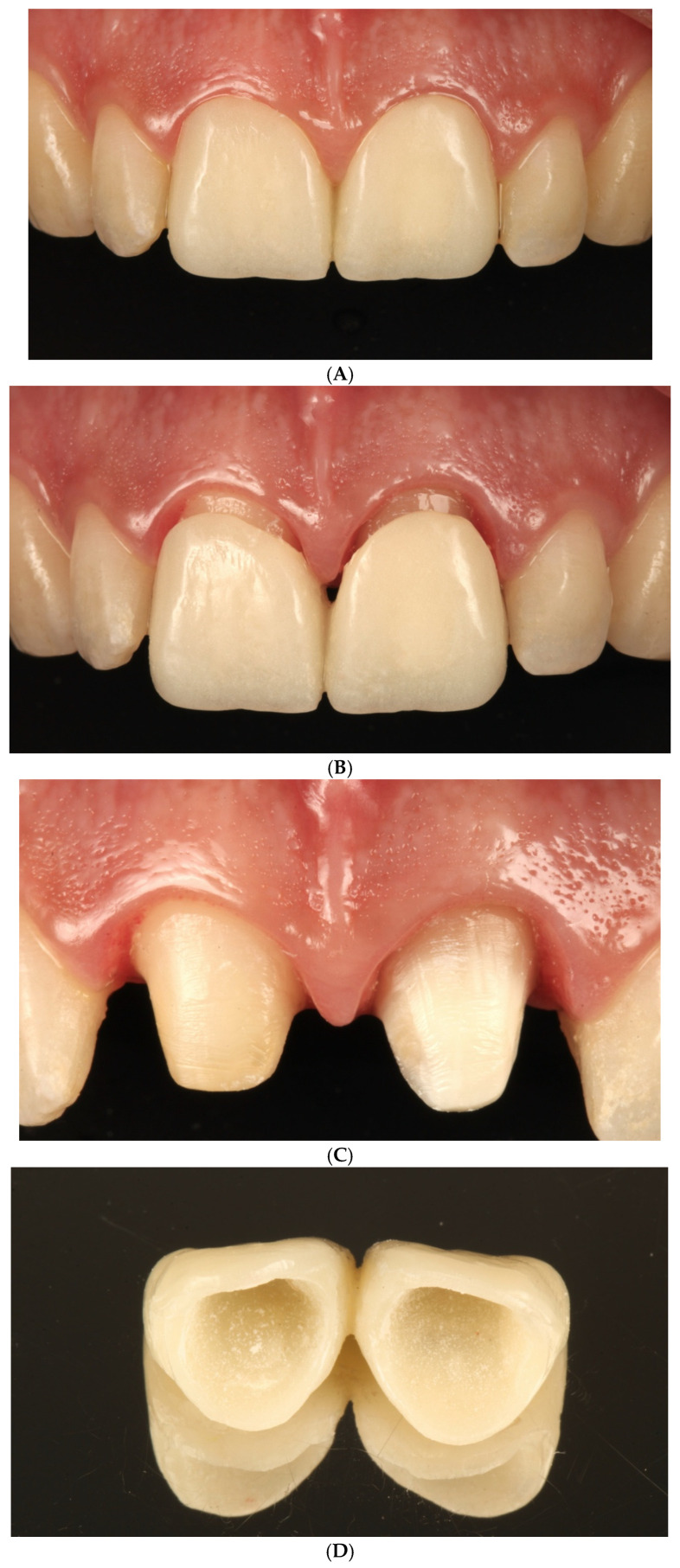
(**A**) Clinical aspect of the gingiva after four weeks of healing with the eBOPT provisional restoration in place. (**B**) Image taken after removal of the eBOPT provisional restoration at four weeks, showing stabilization and initial maturation of the surrounding soft tissues. (**C**) Frontal view of the healed gingiva four weeks after BOPT preparation and use of the eBOPT provisional restoration, with regular gingival contours and healthy tissues. (**D**) Extraoral view of the eBOPT provisional restoration. The image highlights the precision of the line angles and the mirror-like similarity of the cervical morphologies of both provisional restorations. Such an exact duplication can only be achieved when the design of the provisional restoration is performed using CAD CAM technology unlike manual techniques where polishing and hand finishing often introduce minor imperfections and asymmetries between adjacent teeth.

At the second appointment, the bis-acrylic direct provisional was removed from the abutment teeth, the BOPT preparation was performed by eliminating the pre-existing finish line (Figure 7), and the laboratory-fabricated CAD-CAM provisional was internally relined (Sintodent). No manual shaping or polishing of the emergence profile was required, as this had been previously established during the digital design process (Figure 8). This provisional restoration remained in place until gingival stabilization (Figure 9), with no need for future additional adjustments, since the symmetry of the gingival margins had been digitally preplanned in a standarized manner.

**Figure 10:**
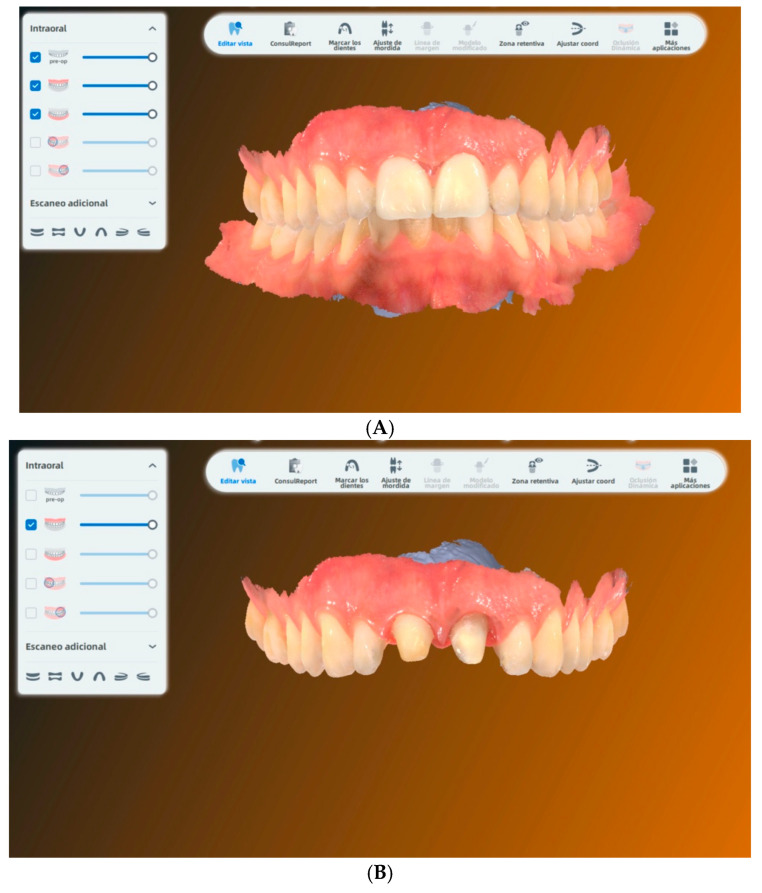

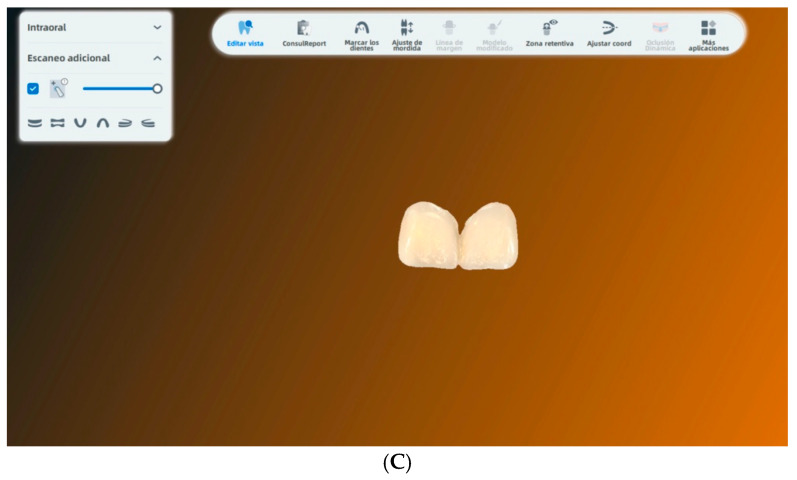
(**A**) Intraoral scan of the eBOPT provisional restoration, corresponding to the first step of the triple-scan protocol and Boolean algorithm execution. (**B**) Scan of the BOPT-prepared teeth and healed surrounding gingiva after removal of the eBOPT provisional restoration. (**C**) Extraoral scan of the eBOPT provisional restoration removed from the abutment teeth, used as the third record in the triple-scan protocol.

**Figure 11:**
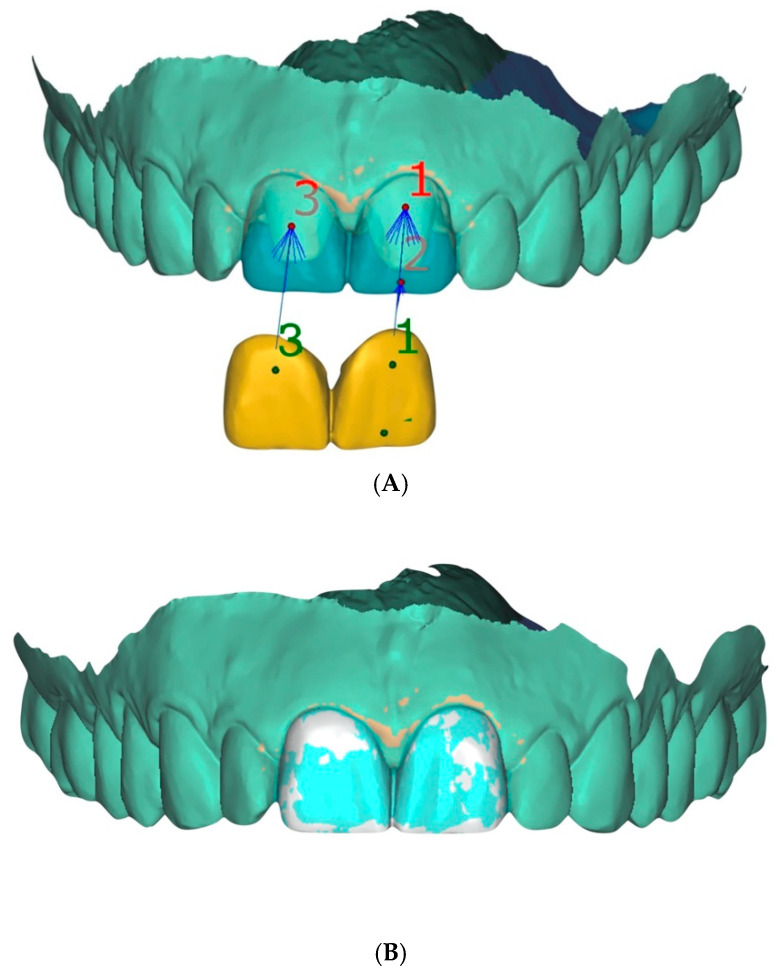

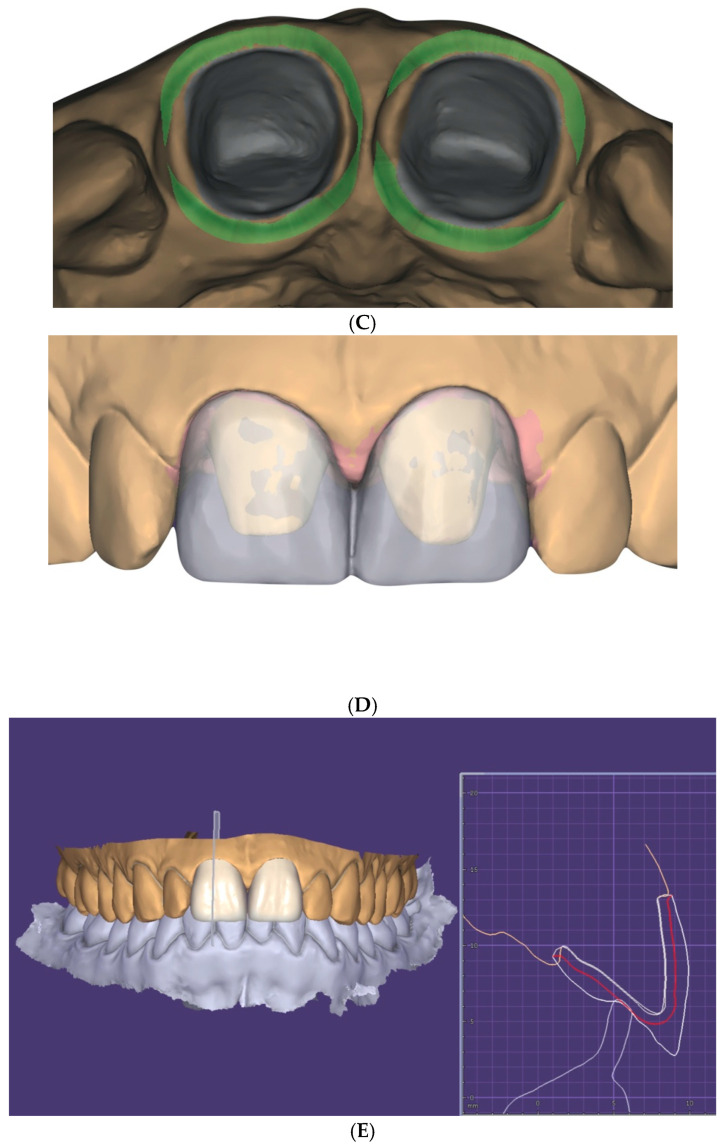
(**A**) Superimposition and alignment (best fit) of the digital scans: the intraoral scan of the cemented provisional, the extraoral scan of the removed provisional, and the scan of the BOPT preparations. (**B**) Complete alignment of the three digital records obtained according to the triple-scan protocol. (**C**) Image of the virtual gingiva generated through the Boolean algorithm, showing in occlusal view the inverse relationship between the gingival contour and the cervical emergence of the extraoral provisional restoration. (**D**) Design of the definitive restorations on the virtual gingiva master model using CAD software, planning zirconia crowns layered with vestibular veneering ceramic. (**E**) Transversal view of the digital design showing the relationship between the definitive restorations and the prepared teeth, as well as the harmonious emergence relative to the virtual gingiva.

After the 12-week healing period, digital scanning was performed to fabricate the definitive restorations. The definitive restoration phase was identical for both groups and was carried out using monolithic zirconia crowns fabricated following the digital triple-scan protocol (Figure 10 and Figure 11) described by Agustín-Panadero [13], enabling the laboratory to accurately duplicate the emergence profile of the provisional restoration and reproduce it in the definitive prosthesis. Before fabrication of the definitive prosthesis, an appointment was scheduled to perform a preliminary evaluation of the fit and occlusion.

**Figure 12:**
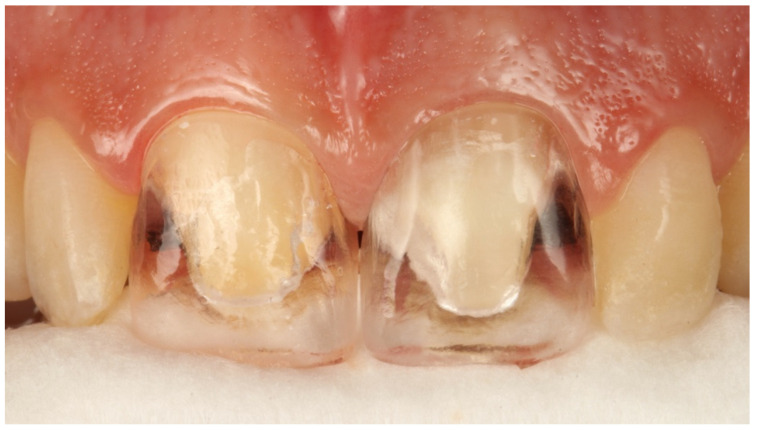
Transparent plastic test for fit evaluation.

**Figure 13:**
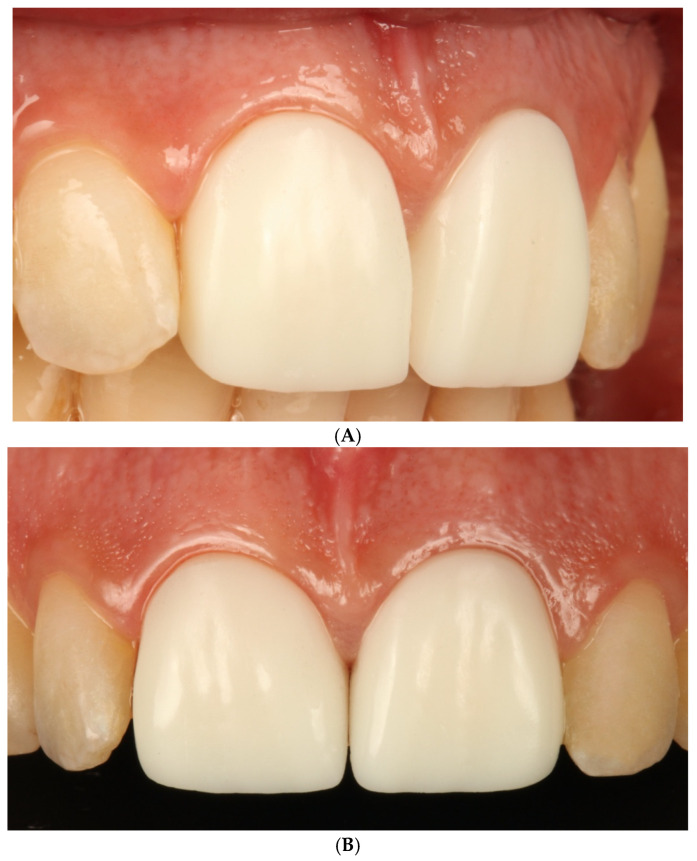
**(A**) White plastic tect for occlusion evaluation. Lateral view. (**B**) White plastic test for occlusion evaluation. Frontal view.

**Figure 14:**
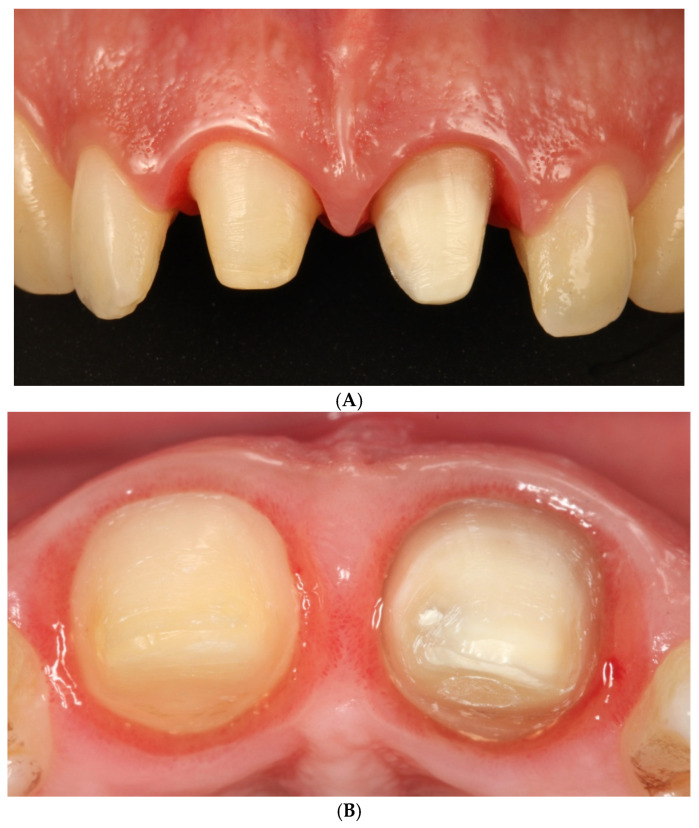
(**A**) Clinical aspect of the prepared teeth after six weeks of healing, before cementation of the definitive restorations. (**B**) Occlusal view of the healed gingiva six weeks after preparation, prior to final cementation of the zirconia restorations.

**Figure 15:**
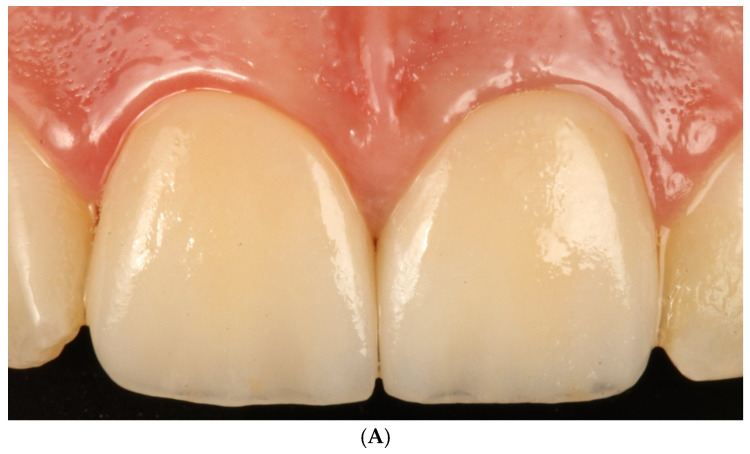

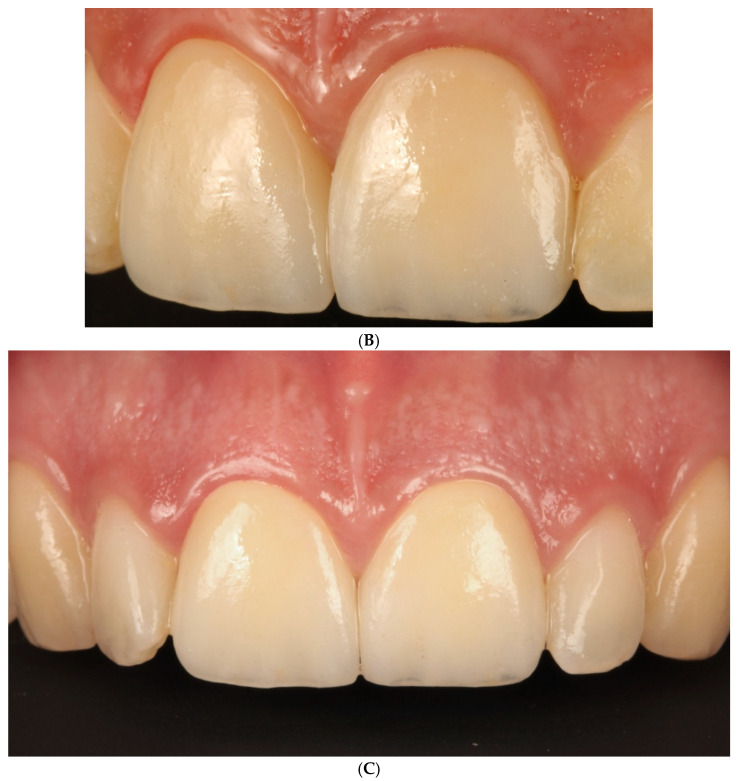
(**A**) Frontal view after cementation of the ceramic restorations on teeth 11 and 21 using composite resin cement. (**B**) Lateral view after cementation of the ceramic restorations. An optimal cervical emergence and harmonious alignment of the gingival margins of teeth 11 and 21 are observed. (**C**) Final image after 12 weeks of complete soft tissue healing.

**Figure 16:**
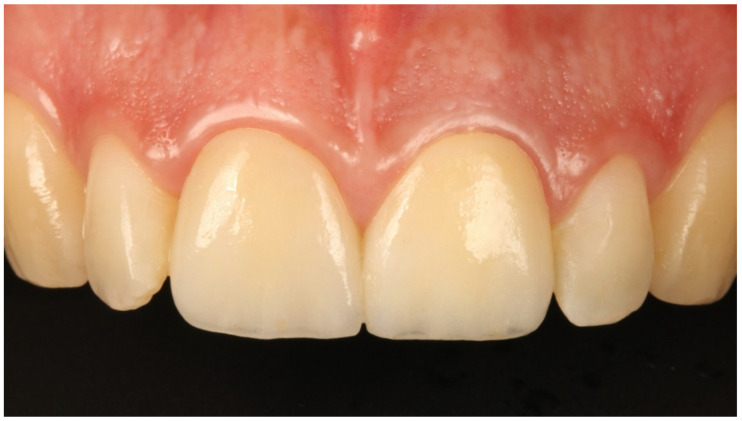
Frontal image after 6 months with the final restorations. Healthy and stable peri-restorative tissues are evident, with full papillary adaptation and esthetic integration of the definitive restorations with adjacent teeth.

**Figure 17:**
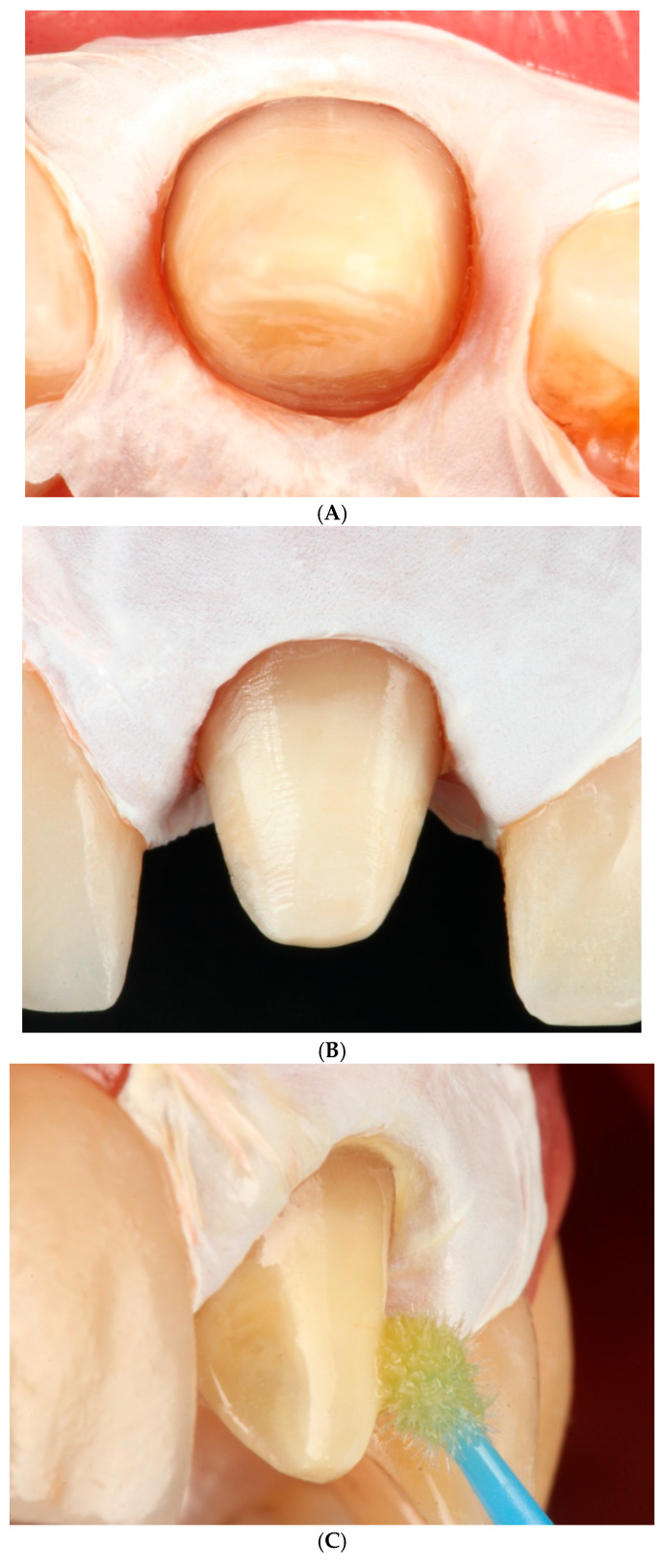

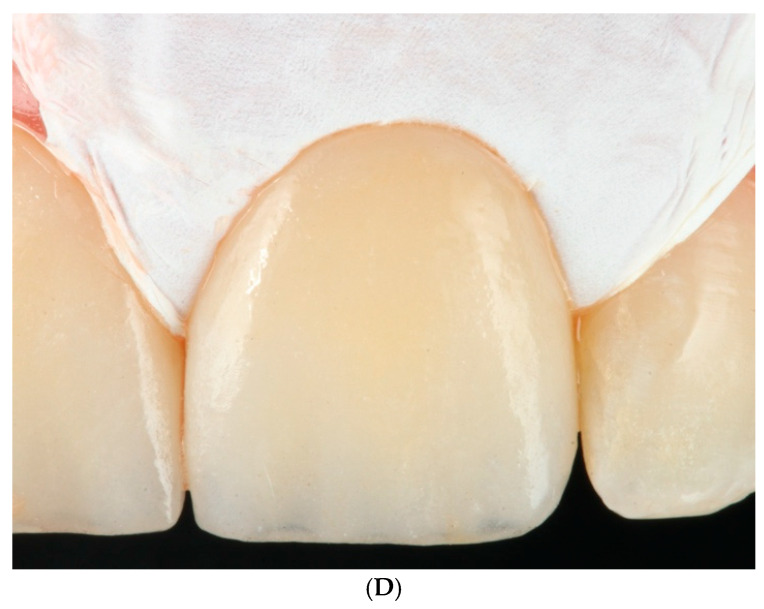
(**A**) Cementation using the relative isolation technique with PTFE tape. Occlusal view of the prepared tooth. (**B**) Cementation using the relative isolation technique with PTFE tape. Frontal view of the prepared tooth. (**C**) Cementation using the relative isolation technique with PTFE tape. Application of the adhesive. (**D**) Cementation using the relative isolation technique with PTFE tape. Cementing crown.

**Figure 18:**
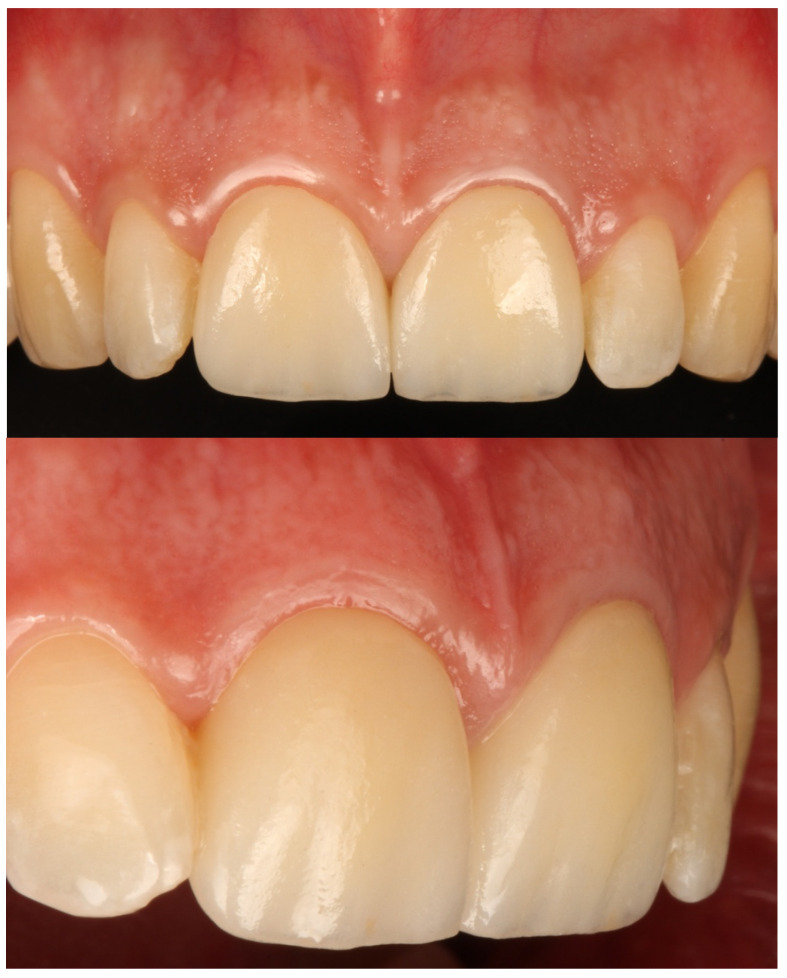
Images, frontal and lateral views, after 12 months of clinical follow-up showing complete circumferential gingival adaptation to the cervical area of the BOPT restorations, with periodontal tissues exhibiting no signs of inflammation.

The fit was assessed using a transparent plastic test (Figure 12), and the occlusion was evaluated using a white plastic test (Figure 13). The definitive restorations (Figure 14, Figure 15 and Figure 16) were cemented with resin composite cement, using the relative isolation technique with PTFE tape as described by Roig-Vanaclocha [15] (Figure 17 and Figure 18).

Standardized periodontal parameters such as gingival index (GI), probing depth (PD), bleeding on probing (BOP) and plaque index (PI) were recorded at baseline and during follow-up visits. During both protocols, the following variables were recorded: total operative time per tooth (defined as the time (in minutes) including only the BOPT vertical preparation and the corresponding provisionalization step (conventional relining and processing or eBOPT relining)), gingival healing time (defined as the number of weeks required to achieve gingival stability, established clinically as the absence of changes in marginal height four weeks apart, without bleeding on probing), and the need for additional adjustments during the healing process (defined as the number of additional appointments required to modify the provisional contour due to gingival remodeling needs like emergence contours, nivelation of gingival margins or presence of microgaps).

Gingival stabilization was defined as the clinical time point at which the gingival index (GI) reached 0 and no bleeding on probing (BOP) was detected. Marginal position changes were assessed through a combined clinical and digital approach. Standardized intraoral photographs were obtained at baseline and at 1, 4, 8, and 12 weeks using consistent magnification and lighting conditions. In addition, digital intraoral scans were performed at the same time points, and the resulting STL files were superimposed using a best-fit alignment algorithm to allow precise comparison of soft tissue contours over time. Marginal changes were measured digitally on the aligned STL datasets using reference points located on stable dental structures to minimize distortion. All measurements were performed twice by the same calibrated operator with a two-week interval between measurements to assess intra-examiner consistency. The mean of the two measurements was used for statistical analysis. This protocol was implemented to reduce measurement bias and improve reliability within the limitations of a case series design.

## 3. Results

The sample consisted of ten patients (6 women and 4 men) with a mean age of 48.2 ± 8.3 years. All participants presented with pre-existing full-coverage restorations in the maxillary anterior region and completed the 12-week clinical follow-up without data loss. The group treated with the conventional BOPT protocol included five patients with a total of 18 restored teeth (n = 18), whereas the eBOPT group included five patients with 17 treated teeth (n = 17). No relevant biological complications or loss of pulpal vitality were observed, and all teeth remained clinically stable throughout the follow-up period.

Baseline periodontal parameters were comparable between groups. Gingival healing was defined as the time point at which the gingival index reached 0 and bleeding on probing was absent. According to this objective criterion, stabilization occurred earlier in the eBOPT group. Probing depth remained stable and plaque levels decreased during follow-up in both groups (Table 1).

**Scheme 3:**
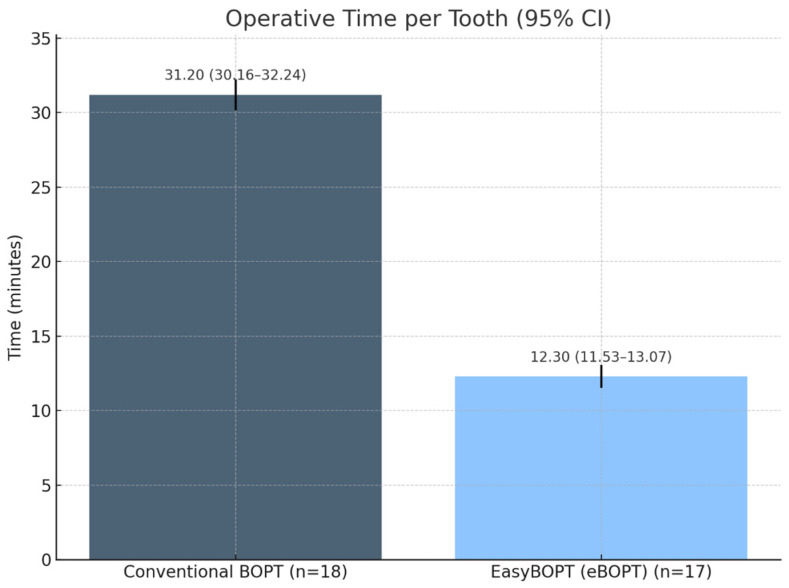
Mean operative time per tooth for both protocols. The eBOPT group showed a significant reduction in total preparation and provisionalization time (*p* < 0.001).

**Scheme 4:**
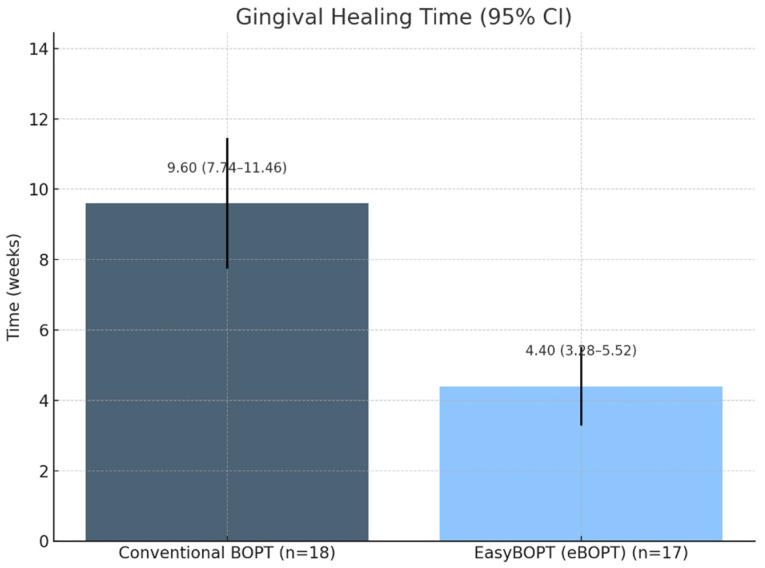
Mean gingival healing time. The eBOPT protocol achieved gingival margin stability in half the time required by the conventional BOPT protocol (*p* = 0.0014).

The mean operative time per tooth including both tooth preparation and provisionalization was lower in the eBOPT group (12.30 ± 1.50 min) compared with the conventional protocol (31.20 ± 2.10 min), with a mean difference of 18.9 min per tooth (*p* < 0.001), representing a clinically relevant reduction in total treatment time (Scheme 3). Likewise, mean gingival healing time was significantly shorter in the eBOPT group (4.4 ± 0.9 weeks) than in the conventional BOPT group (9.6 ± 1.5 weeks), with a difference of 5.2 weeks (*p* = 0.0014), corresponding to a 54.2% reduction in tissue maturation time (Scheme 4) (Table 2).

**Scheme 5:**
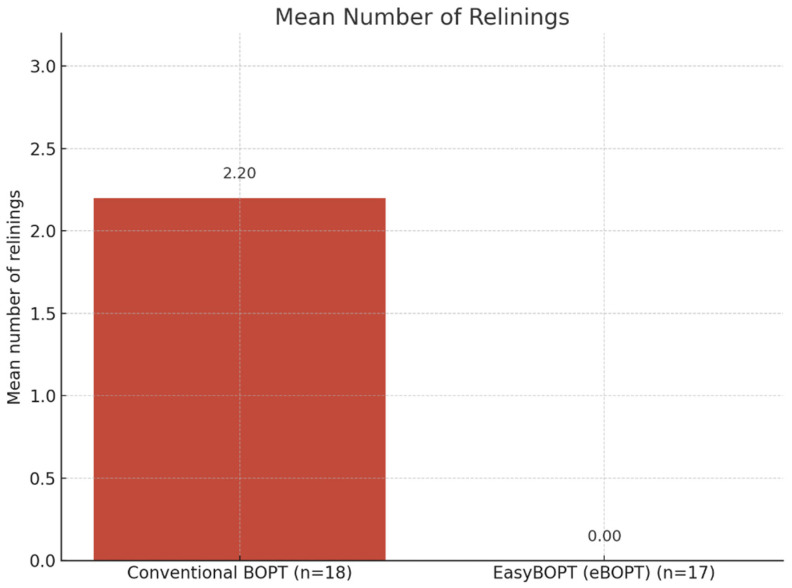
Mean number of additional provisional adjustments. Successive relinings were required in the conventional protocol, whereas the eBOPT protocol completely eliminated this need (*p* = 0.048).

Regarding the need for additional adjustments of the provisional restoration, four out of five patients in the conventional group (80%) required successive relinings (mean: 2.2 ± 0.8 per case), whereas none of the patients in the eBOPT group required any further modification, a statistically significant difference (*p* = 0.048) (Scheme 5). Complete gingival stability was achieved in both groups by the end of the follow-up period; however, in the eBOPT group, it was obtained more rapidly and predictably, generally within 4 to 6 weeks, compared to 8–12 weeks with the conventional protocol.

The CAD-CAM provisionals in the eBOPT group exhibited uniformly polished subgingival surfaces upon insertion, whereas in the conventional group, irregularities and the need for repolishing were occasionally observed. Clinically, patients treated with the digital protocol reported greater comfort and less subjective inflammation during the healing process.

Overall, the findings suggest that the eBOPT workflow may reduce clinical and biological treatment times while maintaining gingival stability comparable to that achieved with the conventional BOPT protocol.

## 4. Discussion

The results of the present study demonstrate that the eBOPT protocol simplifies and standardizes the critical provisionalization phase of the BOPT, leading to a significant reduction in both operative time and gingival healing time, without compromising gingival margin stability. This procedural improvement enhances the clinical applicability of BOPT, already extensively validated in the literature, by reducing operator dependency and increasing reproducibility. The observed reduction in operative time could also contribute to improved chairside efficiency, decreased patient fatigue during anterior rehabilitations, and more flexible scheduling, particularly in multi-unit treatments. However, these potential benefits should be interpreted cautiously given the limited sample size. Similarly, the shorter gingival healing time observed in the eBOPT group should be considered not only statistically but also from a biological standpoint, as earlier tissue stabilization may allow a reduction in the provisional phase and fewer adjustment visits. Nevertheless, confirmation of these clinical implications requires validation in larger controlled studies with longer follow-up.

The conventional BOPT, introduced by Loi [1], is based on a vertical preparation with rotary gingitage aimed at creating a bleeding bed that enables clot stabilization and soft tissue regeneration. The provisional restoration plays a central role in this protocol, acting as a biological mold that guides gingival healing and determines the final position of the margin [16,17,18]. Prospective studies have shown that this approach promotes gingival thickening and notable marginal stability [8,9,11,12,18]. In a two-year clinical trial [8], a mean increase of 0.49 mm buccally and 0.39 mm palatally was reported, with no gingival recessions observed in the treated teeth. Over the long term, a ten-year follow-up study [9] by the same group confirmed the persistence of this effect, showing recessions in only 7.7% of treated teeth compared to 38.4% of untreated antagonists, with a survival rate of 97.8%. These findings consolidate BOPT as a biologically reliable and predictable restorative technique. Nevertheless, the provisionalization phase in the conventional protocol presents significant technical limitations. The “eggshell” provisional restoration must be relined intraorally, and the clinician manually defines the emergence profile through acrylic additions and polishing. This phase, described in detail by Serra-Pastor [11], is critical because it determines the periodontal response; however, it is highly dependent on the operator’s skill. Furthermore, it often requires multiple adjustments over an 8–12-week period until gingival margin stabilization is achieved. Although this process can be successful in experienced hands, it introduces clinical variability and extends the overall treatment time.

The eBOPT protocol represents a natural evolution of the technique, adapted to the digital environment. The key factor lies in the CAD-CAM provisionals, which are digitally designed by superimposing STL files obtained with the gingiva in its physiological and retracted positions. These provisionals incorporate a standardized emergence profile and laboratory-polished subgingival surfaces from the outset. This approach eliminates the need for manual modeling, reduces inter-operator variability, and prevents successive relinings.

The BOPT protocol, particularly in its conventional form, is technique-sensitive and requires a thorough understanding of periodontal biology, precise control of the vertical preparation depth, and, most importantly, an accurate manual design of the provisional emergence profile. Therefore, clinicians with limited experience may observe different outcomes, especially regarding gingival stability and marginal adaptation. In contrast, the EasyBOPT protocol was specifically developed to reduce operator-dependent variability by transferring the critical manual design phase of the emergence profile to a digital workflow. By standardizing the cervical contour design and minimizing manual intraoral modifications, EasyBOPT may improve reproducibility and shorten the learning curve. It is necessary to state that all clinical procedures described in this manuscript were performed by a single experienced operator to minimize technical variability, which represents a limitation of this study as it may affect the external validity of the findings. However, within the EasyBOPT protocol, it is not crucial for the operator to possess advanced manual expertise in provisional fabrication (traditionally one of the most technique-sensitive aspects of the conventional protocol) since the emergence profile is digitally designed and predetermined. Nevertheless, adequate training in diagnosis, biological principles, and digital workflow integration remains essential to achieve predictable results across clinicians with varying levels of experience.

The present study must be interpreted within the limitations inherent to a small prospective case series. Although statistically significant differences were observed, the limited sample size and the inclusion of multiple teeth per patient require cautious interpretation of inferential comparisons. No multilevel statistical modeling was performed to account for potential intra-patient clustering, as the primary objective was protocol description and feasibility assessment rather than definitive comparative effectiveness analysis. Larger randomized controlled trials with appropriate hierarchical statistical models are necessary to confirm these findings. Patient-reported outcomes such as comfort, esthetic satisfaction, and postoperative sensitivity were not systematically, representing a limitation. Future controlled investigations should incorporate validated patient-centered outcome measures to provide a more comprehensive assessment of the clinical impact of the protocol.

The eBOPT protocol preserves the biological foundations of the original BOPT, which have been confirmed by over a decade of clinical evidence, while addressing its primary limitation: operator-dependent manual provisionalization. By integrating a digital workflow, it standardizes emergence design, reduces clinical time, and enhances treatment reproducibility. Overall, it represents a predictable and coherent evolution of the BOPT concept, fully aligned with contemporary trends toward digital and minimally invasive dentistry.

## 5. Conclusions

Within the limitations of this prospective case series, the eBOPT protocol appears to offer a simplified and more standardized provisionalization workflow while preserving the biological principles of conventional BOPT. The digital integration of emergence profile design may reduce clinical time and the need for provisional adjustments, pending confirmation in larger controlled studies.

## 6. Clinical Implications

The eBOPT protocol may represent a promising digital alternative to the conventional BOPT, facilitating reduction in operative time and provisional adjustments while maintaining biological principles, subject to confirmation in future controlled trials.

## Figures and Tables

**Table 1:** Periodontal parameters recorded at baseline and during follow-up in patients treated with conventional BOPT and eBOPT protocols. Values are expressed as mean ± standard deviation (SD).

Parameter	Time Point	Conventional BOPT (Mean ± SD)	eBOPT (Mean ± SD)
**Gingival Index (GI)**	Baseline	1.8 ± 0.4	1.7 ± 0.5
	At gingival stabilization	0	0
**Probing Depth (mm)**	Baseline	2.9 ± 0.6	2.8 ± 0.5
	Final follow-up (12 weeks)	2.4 ± 0.4	2.3 ± 0.3
**Bleeding on Probing (BOP)**	Baseline	Present	Present
	At gingival stabilization	Absent (0%)	Absent (0%)
**Plaque Index (PI)**	Baseline	1.5 ± 0.5	1.4 ± 0.6
	Final follow-up (12 weeks)	0.5 ± 0.3	0.4 ± 0.2

**Table 2:** Comparison of operative time and gingival healing time between the conventional BOPT and eBOPT protocols. Values are expressed as mean ± standard deviation (SD) and 95% confidence interval (95% CI). Comparisons between groups were performed using Welch’s *t*-test for continuous variables.

Variable	Conventional BOPT (Mean ± SD)	95% CI (Conventional)	eBOPT (Mean ± SD)	95% CI eBOPT	Mean Difference	*p* (Welch)
**Time per tooth (min)**	31.20 ± 2.10	[30.16–32.24]	12.30 ±1.50	[11.53–13.07]	18.90	<0.001
**Healing time (weeks)**	9.6 ± 1.5	[7.74–11.46]	4.4 ± 0.9	[3.28–5.52]	5.2	0.0014

## Data Availability

The original contributions presented in this study are included in the article. Further inquiries can be directed to the corresponding author.
